# Teaching webside manner: development and initial evaluation of a video consultation skills training module for undergraduate medical students

**DOI:** 10.1080/10872981.2021.1954492

**Published:** 2021-07-27

**Authors:** Charlotte K. Gunner, Emily Eisner, Angus JM Watson, John L. Duncan

**Affiliations:** a Highlands Medical Education Centre, University of Aberdeen, Inverness, UK; b Division of Psychology and Mental Health, School of Health Sciences, University of Manchester, Manchester, UK AND Research and Innovation Department, Greater Manchester Mental Health NHS Foundation Trust, Manchester, UK; c Department of Colorectal Surgery, NHS Highland, Inverness, UK

**Keywords:** Remote consultation, video consultation, simulation, COVID-19, medical education

## Abstract

**Background:**

Video consultations are increasingly used to communicate with patients, particularly during the current COVID-19 pandemic. However, training in video consultation skills receives scant attention in the literature. We sought to introduce this important topic to our undergraduate medical school curriculum.

**Objective:**

To increase final year medical students’ video consultation skills and knowledge.

**Methods:**

We used Plan, Do, Study, Act (PDSA) quality improvement methodology with a pre-post study design to develop a teaching session for 5th year medical students, informed by a literature review and online clinician survey. The 2 hour session comprised an introduction and three practical stations: patient selection and ethics, technology and example videos, and simulation. Subjective pre- and post-session confidence was reported by students across seven domains using 5-point scales (1: not at all confident; 5: extremely confident). Students and facilitators completed post-session feedback forms.

**Results:**

The 40 students and 3 facilitators who attended, over two separate teaching sessions, provided unanimously positive feedback. All students considered the session relevant. Subjective confidence ratings (n = 34) significantly increased from pre- to post-session (mean increase 1.78, p < 0.001).

**Conclusions:**

The inaugural teaching session was well-received and subjective assessment measures showed improvement in taught skills. This pilot has informed a UK-wide multi-centre study with subjective and objective data collection.

## Introduction

The COVID-19 pandemic has provided an acute catalyst for an exponential rise in the use of video consultations, with remote consultations currently used as the default mode of communication with patients, wherever possible, to reduce the risk of virus transmission [1–6]. These elevated rates of remote consultation will almost certainly continue after the return to more ‘normal’ healthcare provision [7,8]. Nevertheless, it is important to acknowledge that the recently increased *quantity* of remote consultations does not necessarily mean that clinicians are now fully equipped to offer their patients high-*quality* remote consultations.

Concerns regarding video consultation have been well documented, particularly pertaining to cost effectiveness, impacts on health outcomes and whether such technology serves a diverse case mix [9–12]. Since COVID-19 has forced this technology into wider use, guidance has been issued to healthcare practitioners around the world to facilitate the decision making process around provision of remote treatment [13–17]. This guidance centres on ensuring that patients with complex needs have the capacity to engage with consultation, that the consulting practitioner has all the necessary information to hand and that secure and reliable prescribing systems are in place. Such guidance is clearly essential in the short term, while doctors familiarise themselves with the basics of remote consultation. However, in the longer term it will also be crucial to ensure that future clinicians are formally trained to conduct high-quality video consultations.

Newly graduated doctors are expected to be able to communicate effectively with patients, whether when providing care in person or remotely [18]. Nevertheless, at present, the teaching of video consultation skills is far from mainstream [19]. It is therefore of utmost importance that this topic is introduced to the undergraduate curriculum so that those offering future video consultations are trained to provide safe and effective patient care which adheres to national guidelines.

While clinical and practical aspects of the delivery of telemedicine have a stronger presence in the literature, education on delivery of video consultation is minimally represented. Prior to the pandemic only half of American medical schools included telemedicine in their curriculum [20] and since the pandemic there remains a gap in the literature in this area [21]. A single published systematic review from 2017 found only nine studies (published 2008–2014) relating to education and training in ‘clinical telehealth’ [22], many of which did not cover video consultation specifically in their training sessions. Where publications describe specific video consultation skills teaching, results have largely been positive [23,24], although samples are typically small and it is difficult to determine the longer-term impact of the teaching. One pilot study reported that, in the absence of prior training, graduate medical trainees perform poorly on simple assessment and management tasks via video consultation technology [25]. This suggests that training in video consultation is essential prior to use in a clinical environment.

In the words of one narrative review, ‘*medical education has a responsibility to ensure that physicians are prepared to effectively leverage the power of technology to serve their patients*’ [26]. This has never been more relevant than at present, where the use of technology to assist in the delivery of patient care is rapidly increasing. There is an urgent need for further integration of such an important topic into mainstream medical education [20,22].

We therefore sought to add video consultation skills to the undergraduate medical curriculum at our university. To this end, we have developed a teaching programme in skills specific to video consultation. The current paper describes the development and piloting of this teaching programme, in preparation for a large-scale multi-site study across UK medical schools.

## Methods

### Design

**Figure 1.**
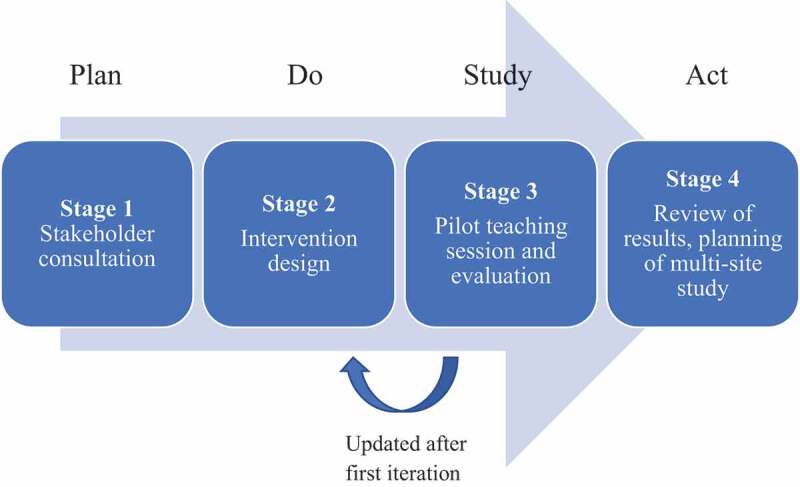
Process map

‘Plan, Do, Study, Act’ (PDSA) quality improvement methodology with a pre-post study design was used to introduce the proposed curriculum changes in a structured and measured way [27]. PDSA was initially outlined in 2009 [27] and focusses on a model for improvement with a cyclical nature: define the objective (plan), carry out the plan (do), analyse the data and summarise the learning (study) and, finally, decide what changes are needed in order to plan the next cycle. A process map for the project is shown in Figure 1.

### Ethics and data protection

Local ethics committee formal exemption from ethical approval was received due to the quality improvement nature of this project. The video consultation software [28] used during the teaching session is compliant with GDPR and the UK Data Protection Act 2018 [29]. Students had no access to real video consultations and simulated consultations were not recorded. All evaluation forms were completed anonymously and stored securely.

### Stage 1: stakeholder consultation (‘Plan’)

Key stakeholders for this project were identified using the ‘9 Cs’ method (commissioners, customers, collaborators, contributors, channels, commentators, consumers, champions, competitors) [30]. Clinicians who currently carry out remote consultations were considered a key stakeholder group, since today’s medical students should be ready to assume such roles in the future. Two local clinicians (consultants in Diabetes/Endocrinology and Gastroenterology) who regularly use the video consultation software NHS Near Me were interviewed by the first author (CKG). These semi-structured interviews followed a topic guide; detailed notes were taken by the interviewer (guide and interview notes available on request).

Interview responses informed the design of an online survey to enable data collection from a wider sample of clinicians with experience of conducting video consultations. The 17-item survey was subsequently distributed to all clinician users of NHS Near Me registered within our organisation (n = 332) in mid-2019, before the COVID-19 pandemic. Survey responses were returned by 45 clinicians (13.5% response rate); 4 responses were excluded due to lack of use of video consultation. The largest group of respondents was medical or surgical consultants (41.5%) followed by specialist nurses (19.5%). 53.7% had been using video consultations for under a year, 14.6% each for 1–2 years, 2–5 years and 5–10 years and a single clinician (2.4%) for longer than 10 years. Median clinician estimate of outpatient workload carried out by video at the time was 8% (range 1–40%).

31.5% of users reported receiving some form of training in skills specific to remote consultation, all at postgraduate level. Those who did receive training mainly learnt about the technical setup (36%) either informally or at an induction from NHS Near Me. Consistent with the published literature [22,24,31], respondents suggested the use of simulation as one of the best ways to introduce video consultation to students and qualified professionals alike. Suggestions for topics to cover in a teaching session included patient selection, confidentiality, support mechanisms for the patient and practical aspects of technical equipment. Communication skills, specifically clear speech, awareness of body language over video, putting patients at ease, verbalising clear management plans, checking of patient understanding and techniques to bring a video consultation to a close were also recommended by respondents. One respondent suggested playing a recording of an exemplar video consult to form the basis of a discussion of the above points, which has been shown to be effective in the literature [23].

### Stage 2: intervention design (‘Plan’)

Seven key areas were identified from the published literature and stakeholder consultation as important focus points for an intervention. These formed the learning outcomes (Textbox 1), which were designed according to Bloom’s principles which comprise a hierarchical model designed to classify educational learning objectives [32]. These provided a framework around which the educational intervention was carefully designed. An active learning approach was taken, as this aligned well with the proposed learning outcomes and has been shown to increase student performance, particularly in small group settings with fewer than 50 learners [33].

Final year medical students were chosen as the recipient learners since their schedules place them in clinical practice for longer periods of time than other year groups, increasing their chances of encountering video consultation in practice and thus the perceived relevance of the teaching on their future careers.

For the first iteration of the PDSA cycle, the intervention was designed as a single 2 hour teaching session for around 20 students, comprising a short introduction for the entire group, maximising the height of attention in group teaching during the first 10–20 minutes [34], followed by a carousel of three teaching stations, using a similar structure to that published by Rienits et al [23]. The session was slightly adjusted for its second iteration based on the evaluation findings; it was lengthened to 2 ½ hours and the rotating carousel condensed into two stations without altering content.

**Textbox 1.** Learning outcomes

By the end of the programme students should be able to: Define remote consultation and identify commonly used alternative terms such as telemedicine, e-health and video consultation Identify patients suitable for video consultation Describe the consent process for video consultation Describe technical and procedural issues arising within video consultations Describe the key elements of a safe and effective video consultation Demonstrate assessment of a patient using video consultation Discuss ethical issues surrounding the process of video consultation

#### Teaching session outline

**Figure 2.**
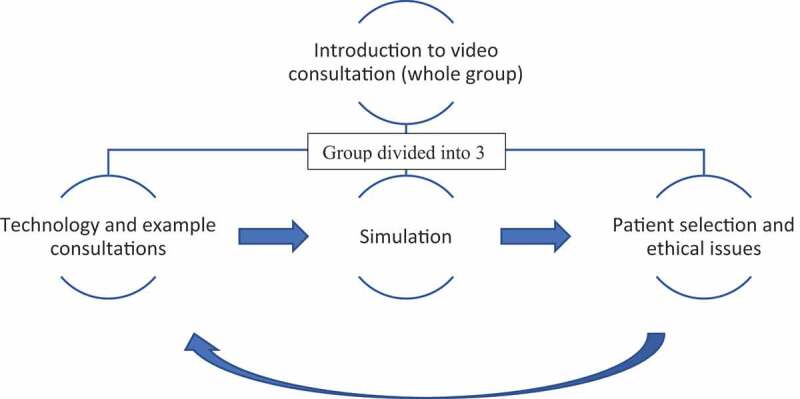
Teaching session overview

An overview of the teaching session structure is shown in Figure 2.

Introduction to video consultation (20 minutes)

- Definitions of common alternative terms such as telehealth and remote consultation
- A brief history of its use in healthcare
- Interactive discussion of its advantages and disadvantages

The group then rotated round three interactive teaching stations, each lasting 30 minutes, facilitated by a member of teaching staff (referred to henceforth as a ‘facilitator’) with experience of video consultation. The three stations were:

Technology and example consultations*

- Practical session looking at the setup of a video consultation using NHS Near Me video consultation software [28] including an appropriate backdrop, lighting, sound and camera position
- Discussion of the structure of a consultation and how this differs when using video compared to face-to-face
- Example videos of two video consultations, filmed with actors, one ‘good’ and one ‘bad’. These were made based on the guidelines and stages 1 and 2 of the study. They were used as a way to illustrate the principles covered in the teaching session and carefully scripted to do so. These example videos can be accessed online via the links in the supplemental material

Simulation*

- Use of a simulated patient to allow students to take a history or discuss a management plan using NHS Near Me video consultation software [28]
- Students also given the opportunity to ‘be’ the patient to appreciate the differences of a consultation from both sides when using video

Patient selection and ethical issues

- Selection of mock patient summaries for students to group according to suitability for video consultation
- Facilitated discussion of potential algorithms to assist in patient selection
- Discussion of potential ethical issues around the use of video consultation, including issues surrounding data storage and confidentiality
- Discussion and examples of protocols for video consultation, including when things go wrong
- Discussion of consent process for video consultation

*combined into a single teaching station for the second iteration

### Stage 3: pilot teaching session and evaluation (‘Do’ and ‘Study’)

The pilot teaching session was carried out on two separate occasions with all teaching delivered in-person, with the exception of the simulated video consultation which was carried out using a remotely located volunteer patient.

In line with quality improvement methodology using the PDSA cycle, students’ baseline knowledge was measured [35]. Prior to the start of the session, students completed a self-report questionnaire, rating their confidence on a 5-point scale from 1 (not at all confident) to 5 (extremely confident) regarding their ability to fulfil each of the seven learning outcomes (questionnaire available in supplemental materials). The same questions were asked again at the end of the session. Pre- and post-session self-reported confidence scores across the seven learning outcomes were compared using paired sample t-tests. In addition, students were asked the level (1–5, strongly disagree – strongly agree) to which they agreed with a series of statements relating to the relevance and quality of each of the three stations of the teaching session.

While this was not a pre-validated questionnaire, it was carefully designed based on the four dimensions of target parameters described by Gibson et al in their validated, structured approach to evaluation of teaching [36]: structural, procedural, teacher characteristics and outcome of teaching activities. There is significant overlap between these four domains and the nine domains described by Marsh in the widely used Students’ Evaluations of Educational Quality (SEEQ) [37].

Facilitators were also asked to complete evaluation forms asking six open questions about their experience of facilitating the sessionfor example, how the session could be improved and suggestions for other areas to be covered in the teaching session in the future.

### Stage 4: planning of multi-site study (‘Act’)

Planning is currently underway for a multi-site study with recruitment of medical schools across the UK ongoing. Teaching materials developed during the pilot study have been refined and adjusted to allow online delivery during COVID-19 restrictions and will be distributed to participating centres. Participating centres will collect data on subjective and objective assessment of student knowledge and skills before and after the delivery of video consultation skills teaching, building on methods developed during this pilot study.

## Results

### Stage 3 pilot teaching session

Two face-to-face teaching sessions were provided to a total of 40 final year medical students. 34 students completed evaluation forms. Subjective feedback was unanimously positive. All students perceived an increase in their confidence at fulfilling the learning outcomes following the session, when compared to pre-session self-rating scores, an effect that was statistically significant (p < 0.001) across all learning outcomes (see Table 1).

**Table 1.** Student self-assessment of confidence against learning outcomes pre- and post-teaching, where scores range between 1 (not at all confident) and 5 (extremely confident)

Learning outcome	Pre-session score (n = 34)		Post-session score (n = 34)		Comparison of pre and post session scores		
	Mean	Standard deviation	Mean	Standard deviation	Difference in means	t	p
Define video consultation and identify commonly used alternative terms	2.79	0.76	4.26	0.44	1.47	9.57	<0.001
Identify patients suitable for video consultation	2.44	0.77	4.03	0.38	1.59	11.28	<0.001
Describe the consent process for video consultation	2.03	0.89	4.41	0.49	2.38	15.64	<0.001
Describe technical and procedural issues arising within video consultations	2.26	0.70	4.24	0.60	1.97	15.15	<0.001
Describe the key elements of a safe and effective video consultation	2.26	0.70	4.24	0.49	1.97	13.77	<0.001
Demonstrate assessment of a patient using video consultation	2.32	0.83	3.97	0.38	1.65	13.09	<0.001
Discuss ethical issues surrounding the process of video consultation	2.65	0.76	4.09	0.45	1.44	10.20	<0.001

A summary of pre- and post-teaching responses is shown in Table 1. Before the start of the teaching session, student confidence was similar for each of the learning objectives, with a mean score of 2.39 (out of 5) across learning objectives. Students felt least confident describing the consent process for video consultation and most confident defining video consultation and other commonly used terms. By the end of the session, mean scores for all learning objectives had increased substantially (mean across learning objectives 4.18). No student had a fall in confidence in any area at the end of the session. The greatest score increase was seen in the area rated as least confident prior to the session – the consent process for video consultation. The lowest increase in confidence was in ethical issues surrounding video consultation.

All areas of the teaching were felt to be relevant, interactive and led by enthusiastic facilitators. Areas of commendation included the interactive nature of the session, the relaxed environment and the interest of what was perceived to be a relevant subject that had not been touched on before in the students’ education. Numerical student feedback for each individual station was fairly homogenous with all stations scoring over 4.5 out of 5 for each statement. Areas for improvement from the students suggested after the first iteration focussed on timing and provision of simulation; these changes were adjusted for the second iteration.

Facilitator evaluation mirrored that of the students; they liked the interactive design of the session, commenting that the mixture of topics and practical elements worked well and that this was a much-needed addition to the curriculum. Their suggestions for improvement also reflected those of the students, focussing on more time for simulation to allow more students to be directly involved.

## Discussion

This project was undertaken prior to the COVID-19 pandemic, as video consultation was becoming an increasingly used method of communicating with patients in our rural area. However, the advent of the pandemic has caused a surge in the use of video consultation and therefore the relevance of this pilot study. We developed a novel teaching session to increase final year medical student’s skills and knowledge regarding video consultation, targeting an important gap in the curriculum and aligning student learning with national guidelines. Analysis of completed student and facilitator evaluation forms showed positive results, with all agreeing that the session was of benefit. There are, however, several areas that can be improved upon and these formed the focus of the ‘study’ and ‘act’ parts of PDSA.

Student confidence scores increased by the largest margin when referring to the consent process for video consultation. The topic of consent relating to video consultation featured in each of the three small group sessions; this may have allowed consolidation of knowledge through repetition, often historically cited as an essential part of associative learning [38,39]. The smallest increase in student confidence was in ethical issues surrounding video consultation. This was covered in the same small group session as patient selection. Feedback from the facilitator suggested that the balance of time allocation was weighted too heavily towards patient selection, resulting in only brief discussion of ethical issues which may explain these results. This will be adjusted for the next iteration of the session.

Lack of time is mentioned repeatedly in the feedback, largely relating to the simulation session. The design of this small-group session allowed three students to take part in the simulation, however, with group sizes averaging 7 students, this left more than half the group merely observing. Following discussion with facilitators, some solutions have been identified: firstly, the entire teaching session could be made slightly longer to allow a little more time in each of the three small-group rotations. Alternatively, the role of the patient could be played by a student, allowing more students to have a role in simulation. However, the value of volunteer patients in the education of medical students is well documented [40] and it would seem counterproductive to lose this. Simulation scenarios could also be sub-divided, and students substituted in, or more time allocated to the simulation aspect to allow addition of a third simulation scenario.

There were some logistical challenges to running this session, mainly involving the number of rooms required to accommodate each breakout session. The advent of social distancing in the COVID era has prompted the adaptation of the teaching session for online delivery for the next iteration of the project, providing a solution to this issue. Online ‘breakout rooms’ are widely available on video conferencing software which may in turn allow more students to take an active role in the simulation element of the session, provided sufficient numbers of volunteer patients are trained and available to participate.

Continuing to follow a plan consistent with Quality Improvement methodology, the next step is to spread the intervention to other sites, described by Massoud et al [41] as ‘an often forgotten stage in an improvement plan’. A nationwide scale-up of this project is currently underway, with recruitment from medical schools across the UK ongoing. Building on this pilot project, the next phase will include baseline and post-teaching objective data collection for all participating students.

### Limitations

Objective evidence of improvement is lacking from this pilot project. The original study protocol did include a video consultation question in an Objective Structured Clinical Examination (OSCE) before and after the teaching session. An existing history taking OSCE station was adapted to be used in a simulated video consultation, with specific marks available for video consultation specific skills. However, due to a Wi-Fi malfunction, the volume of objective data gathered following the teaching session was too small to be meaningful and has hence been excluded from this project summary. The next iteration of the project will include an online pre- and post-teaching objective assessment using a mock video consultation.

Numbers studied in this pilot project are low; while statistical significance was reached, these results should be interpreted with this in mind. Moreover, this study was carried out in a UK institution. With adaptation, however, we feel the contents are highly relevant for healthcare settings around the world using video consultation.

There are well documented arguments surrounding the use of Likert scales [42]. Within the limits of time and feasibility for the scale of this project it was felt that Likert scales were the most appropriate measure to use for data collection in this case.

## Conclusions

The aim of the project was *‘to increase knowledge and skills relating to video consultation in final year medical students*’ and was undertaken as a quality improvement project, using the PDSA methodology. This study addressed a significant gap in the literature relating to education in video consultation: there remain very few papers specifically addressing the education of healthcare professionals in skills specific to video consultation. The current lack of published studies leaves a shortfall between the published guidelines on remote consultation and the foundations on which education is built to meet such guidelines. In order to bridge this gap and fulfil the project aims, we combined literature review, stakeholder consultation and a firm foundation in education theory to develop high-quality educational materials suitable for use with undergraduate medical students. The subjective evidence from this pilot project suggests that this aim was achieved, in the short-term at least. The use of quality improvement methodology, although not often used in medical education, proved a useful structure for such a project.

Further subjective and objective evidence will be gathered from an ongoing UK-wide project based on this initial pilot.

## Supplementary Material

Supplemental Material

## Practice points

- Video consultation skills are now a requirement for all graduating medical students
- Education and training in video consultation skills is not currently prominent in undergraduate medical education
- We have developed a teaching module aiming to improve knowledge and skills of medical students in video consultation
- Our pilot data suggests that this teaching was well received and achieved its primary objectives
- Further work is ongoing with UK medical schools to disseminate this teaching on a national scale
