# Supplementary material for: Teaching webside manner: development and initial evaluation of a video consultation skills training module for undergraduate medical students
Source: Med Educ Online. 2021 Jul 27;26(1):1954492. doi: 10.1080/10872981.2021.1954492 (PMC8317946; doi:10.1080/10872981.2021.1954492)
Supplement: Supplemental Material [file ZMEO_A_1954492_SM5257.docx]

## Supplemental Material

# Video consultation teaching: student evaluation

## Before the session:

| I feel confident in my ability to | *Not at all confident* |  | *Neutral* |  | *Extremely confident* |
| --- | --- | --- | --- | --- | --- |
| Define video consultation and identify commonly used alternative terms | 1 | 2 | 3 | 4 | 5 |
| Identify patients suitable for video consultation | 1 | 2 | 3 | 4 | 5 |
| Describe the consent process for video consultation | 1 | 2 | 3 | 4 | 5 |
| Describe technical and procedural issues arising within video consultations | 1 | 2 | 3 | 4 | 5 |
| Describe the key elements of a safe and effective video consultation | 1 | 2 | 3 | 4 | 5 |
| Demonstrate effective assessment of a patient using video consultation | 1 | 2 | 3 | 4 | 5 |
| Discuss ethical issues surrounding the process of video consultation | 1 | 2 | 3 | 4 | 5 |

## After the session:

| I feel confident in my ability to | *Not at all confident* |  | *Neutral* |  | *Extremely confident* |
| --- | --- | --- | --- | --- | --- |
| Define video consultation and identify commonly used alternative terms | 1 | 2 | 3 | 4 | 5 |
| Identify patients suitable for video consultation | 1 | 2 | 3 | 4 | 5 |
| Describe the consent process for video consultation | 1 | 2 | 3 | 4 | 5 |
| Describe technical and procedural issues arising within video consultations | 1 | 2 | 3 | 4 | 5 |
| Describe the key elements of a safe and effective video consultation | 1 | 2 | 3 | 4 | 5 |
| Demonstrate effective assessment of a patient using video consultation | 1 | 2 | 3 | 4 | 5 |
| Discuss ethical issues surrounding the process of video consultation | 1 | 2 | 3 | 4 | 5 |

## For the individual stations:

| Technology and example consultations | Strongly disagree | |  | |  | | Strongly agree | |
| --- | --- | --- | --- | --- | --- | --- | --- | --- |
| The session was relevant to my future career | 1 | 2 | | 3 | | 4 | | 5 |
| The teaching materials/ equipment provided helped me to learn | 1 | 2 | | 3 | | 4 | | 5 |
| The session was interactive | 1 | 2 | | 3 | | 4 | | 5 |
| The facilitator was enthusiastic | 1 | 2 | | 3 | | 4 | | 5 |

| Patient selection and ethics | Strongly disagree | |  | |  | | Strongly agree | |
| --- | --- | --- | --- | --- | --- | --- | --- | --- |
| The session was relevant to my future career | 1 | 2 | | 3 | | 4 | | 5 |
| The teaching materials/ equipment provided helped me to learn | 1 | 2 | | 3 | | 4 | | 5 |
| The session was interactive | 1 | 2 | | 3 | | 4 | | 5 |
| The facilitator was enthusiastic | 1 | 2 | | 3 | | 4 | | 5 |

| Simulated consultation | Strongly disagree | |  | |  | | Strongly agree | |
| --- | --- | --- | --- | --- | --- | --- | --- | --- |
|  |  | |  | |  | |  | |
| The session was relevant to my future career | 1 | 2 | | 3 | | 4 | | 5 |
| The teaching materials/ equipment provided helped me to learn | 1 | 2 | | 3 | | 4 | | 5 |
| The session was interactive | 1 | 2 | | 3 | | 4 | | 5 |
| The facilitator was enthusiastic | 1 | 2 | | 3 | | 4 | | 5 |

| - What did you like the most about today’s session? | What did you like least about today’s session? |
| --- | --- |
| - How could we improve the session? | Any other comments? |

## Example videos

‘Good’ example: <https://youtu.be/tRKJPD_2s5w>

‘Bad’ example: <https://youtu.be/Hly_3qUDU7s>
